# Blood pressure control and burden of treatment in South African primary healthcare: A cross-sectional study

**DOI:** 10.4102/phcfm.v11i1.2110

**Published:** 2019-12-19

**Authors:** Kevin Pender, Olufemi Omole

**Affiliations:** 1 Division of Family Medicine, Faculty of Health Sciences, University of the Witwatersrand, Johannesburg, South Africa

**Keywords:** Blood Pressure, Control, Burden of Treatment, Primary HealthCare, Issues around medication regimen, navigating healthcare system

## Abstract

**Background:**

Poor blood pressure (BP) control has been associated with high burden of treatment (BOT) in several settings. It is not known whether this relationship holds true for South African primary care.

**Aim:**

The aim of this study was to assess BOT and determine its relationship with BP control amongst patients with hypertension in a large community health centre, south of Johannesburg.

**Setting:**

The setting of this study was carried out in the OPD of Johan Heyns Community Health Center.

**Methods:**

A cross-sectional study involving 239 patients with hypertension was carried out. Information on sociodemography and BP readings in the last 3 months were extracted from patient medical records. A researcher-administered treatment burden questionnaire was also used to collect information on participants’ perceptions of BOT relating to medication regimen, navigating the healthcare system and life style changes and/or social and/or financial issues. Total BOT (TBOT) was determined as the sum of the scores in the three components and categorised as 1–45 = low, 46–90 = moderate and 91–140 = high. Analysis included descriptive statistics and test of association.

**Results:**

Most participants were white (54.2%), > 55 years (52.9%), female (60.1%), married (56.3%), had grade 12 or more education (71.9%) and had no comorbidity (56.7%). The mean duration of hypertension treatment was 113.8 months and most participants were uncontrolled (60.1%). Most participants (75%) reported a low TBOT score, with a mean of 19.7. Amongst participants with clinical comorbidities, most (66.3%) did not consider hypertension to be more burdensome than other comorbid illnesses. There was no significant association between TBOT and BP control (*p* = 0.53). However, participants with a high BOT relating to medication regimen were significantly more likely to be uncontrolled (*p* = 0.04).

**Conclusion:**

Total BOT is low amongst study participants and has no significant influence on BP control. However, improvements in BP control in South African primary healthcare may be hinged on interventions that address problems associated with hypertension medication regimen.

## Introduction

Hypertension remains the most common cardiovascular risk factor^1^ and according to the WHO report of 2013, developing countries, especially sub-Saharan Africa, are at the epicentre of this epidemic, with 46% of adults in this region having the disease.^2^ In South Africa, an estimated 46% of women and 44% of men above 15 years of age have hypertension.^3,4^

Despite the availability of pharmacological treatments and access to health facilities, blood pressure (BP) control rates remain low globally and in South Africa only 24.5% – 57.0% of patients with hypertension are reportedly controlled.^4,5,6,7,8,9,10,11^ The reasons for poor BP control in sub-Saharan Africa are poorly understood and involve multiple, complex and varying factors depending on setting.^7^ This often includes a high burden of treatment (BOT).^12^

Burden of treatment is described as the tasks patients are required to perform to achieve optimal outcomes in the management of their diseases and the effects these on their functionality and well-being.^13,14^ Studies outside of South Africa have reported that high BOT is associated with poor chronic disease control, including hypertension.^13,14,15,16^ A high BOT therefore has potential for adverse outcomes both for the patient and the healthcare system,^13,14,15,16^ such as poor patient adherence to treatment and health advice,^17,18,19^ poor disease control,^14,19,20^ increased hospitalisation,^21,22^ increased mortality and morbidity, increased costs of healthcare,^15,16^ and low quality of life.^23,24^

In South African primary healthcare (PHC), most patients with chronic diseases of lifestyle have hypertension, and in Sedibeng District more than 60% present with the disease.^25^ Despite this, no local study provides information on the magnitude of BOT and the effect of high BOT on the prevalent poor BP control amongst patients with hypertension. Knowing this is useful for developing effective and context-specific interventions for improving BP control in South African PHC. The aim of this study was therefore to assess the magnitude of BOT and determine its relationship with BP control among patients on treatment for hypertension at a large PHC facility in Sedibeng District, Gauteng province.

## Methods

### Study design

A cross-sectional study was carried out.

### Study site

This study was conducted at the outpatient department (OPD) of Johan Heyns Community Health Centre (CHC), Vanderbijlpark, south of Johannesburg. This facility has a large number of patients, cutting across all racial and educational profiles. Apart from catering for Vanderbijlpark, it receives feeders from adjacent farming areas. It serves a total population of 74 075 and provides curative, preventative, antenatal, chronic and rehabilitative care, and basic radiological services.

### Sample size and sampling

The samples were selected from the 627 patients who had attended the facility for hypertension treatment between November 2016 and March 2017. Assuming a confidence interval of 95%, an expected frequency of 50% and confidence limits of 5%, the sample size, calculated using Epi info 7^TM^, was determined to be 239.

In recruiting samples, health promoters addressed patients each morning in the waiting hall about the purpose of the study and informed them that patients who chose to participate in the study would be placed back in the order of their initial position in the queue to see a doctor. Patients who agreed to participate received an information sheet in the vital signs room, and if they met the inclusion criteria, they proceeded to a private room to meet the researchers. Inclusion criteria for the study were as follows: a patient had to be an adult above 18 years of age, must consent to participate in the study, must have attended the clinic for at least 3 months and should be on anti-hypertensive medication.^23^ Exclusion criteria were as follows: if they were only on lifestyle modifications, hypertensive emergencies and unable to comprehend the treatment burden questionnaire (TBQ) despite adequate explanation. In a private room, the researchers repeated the summary of the study purposes to each participant and elicited their understanding. Patients who responded were also informed that full details were on the information sheet they received, and that there were numbers to call or emails to write to, if any questions arose. Finally, consenting patients were aided to fill the consent form. The above routine was followed throughout the period of recruitment. Consecutive sampling was continued until the desired sample size was attained. Participants were recruited Monday to Friday between 08:00 and 16:00.

### Measurement tools and data collection

Two medical doctors who provided usual care in the OPD recruited participants and administered the measurement tools. Three tools were used for data collection: participant characteristic form (PCF), TBQ and hypertension control form (HCF).

The PCF was adapted from the participant tools used in two previous studies in the same district^25,26^ and collected information on age, sex, race, marital status, educational level, time from diagnosis of hypertension to present date, if any other comorbidity and if hypertension was more problematic. The researchers completed the PCF for each study participant.

The TBQ obtained information from participants in three major areas: burden related to medication, burden related to navigating the healthcare system and burden related to lifestyle changes and social and financial impacts.^24^ The TBQ was explained to each participant, including the interpretation of its visual analogue scale: participants were asked to score zero if a question posed no issue or problems to them, to score between 1 and 5 if a question was a moderate problem or posed issue to them and to score between 6 and 10 if a question posed a big issue or problem to them. As deemed necessary by the researcher, each question was explained or clarified to participants before they were scored. Questions that were not applicable to patient’s circumstances were marked as so.

The HCF was developed by the researcher, assisted by the supervisor, and recorded BP readings of the current and last two clinic visits. Blood pressure measurements at visits were routinely performed by trained staff nurses using the oscillometric method of fully automated and calibrated electronic CONTEC^TM^ Patient Monitor machines that are routinely recalibrated every 3 months. The South African Hypertension Society guidelines were followed for measuring the BP.^27^ Finally, the BP for the current and two previous consecutive visits was extracted from participant medical records and entered onto the HCF.

### Analysis

Each consecutive participant received a unique code from 001 to 239 that was placed on the completed TBQ, PCF and HCF to provide a link amongst these tools.

Data from these tools were uploaded into Epi Info 7^TM^ and analysed by the researchers. Participant characteristics were analysed with descriptive statistics. The mean systolic BP (SBP) and diastolic BP (DBP) were determined for the current visit and the visits in the last 3 months. The proportions of participants with BPs controlled to target at current and in the last 3 months were determined according to targets in the 2014 South African hypertension guideline.^27^ The proportions of participants with comorbidities, as well as those who considered hypertension more problematic than other comorbidities, were also determined.

The BOT was determined as follows: the sums of participants’ scores for each question in each of the three sections of the TBQ were recorded as:

- BOT 1: Burden relating to issues of medication regimen.
- BOT 2: Burden relating to navigating the healthcare system.
- BOT 3: Burden relating to lifestyle changes and accessing social/financial support, respectively.

The maximum scores in each of the three sections were divided into three tertiles:

- BOT 1: 1–13 (low BOT), 14–26 (moderate BOT), 27–40 (high BOT).
- BOT 2: 1–16 (low BOT), 17–32 (moderate BOT), 33–50 (high BOT).
- BOT 3: 1–16 (low BOT), 17–32 (moderate BOT), 33–50 (high BOT).

The total BOT score was also determined as the sum of the scores in BOT1, BOT2 and BOT3, and divided into three tertiles and classified as low, moderate and high total composite BOT:

- Total BOT (TBOT): 1–45 (low), 46–90 (moderate), 91–140 (high).
- (*The calculated ranges for BOT 2 and 3 are similar because they contain equal number of questions.)

Analysis of variance (ANOVA) was used to determine if there were significant differences in mean SBP, mean DBP and BOT/TBOT across sociodemographic groups. Regression analysis was used to explore the relationship between age, race, sex, marital status, educational level and treatment duration (explanatory variables) and each of the 15 questions on the TBQ, mean SBP and DBP, BP control to target, BOT1, BOT2, BOT3 and TBOT (dependent variables). Statistical significance was set at *p* < 0.05.

### Ethical considerations

Individual patients gave written informed consent before enrolment in the study. Participants were allocated codes and no personal identifier was used. All data were kept locked in a secure drawer and were accessible only to the research team. Ethics clearance was obtained from the Human Research Ethics committee (medical) at the University of the Witwatersrand (clearance number: M160804) and written permission to conduct the study in the facility was obtained from the Sedibeng District Health Services management.

## Results

A total of 239 participants were enrolled. Most of the participants were white (54.20%), female (60.08%), aged 45–64 years (54.62%), married or cohabiting (56.30%) and had completed high school or more education (71.85%) (Table 1).

**TABLE 1:** Participants’ sociodemographic characteristics (*N* = 239).

Variable	%	*n*
**Age (years)**		
25–34	5.04	12
35–44	11.76	28
45–64	54.62	130
65–74	22.27	53
75–84	6.30	15
**Race**		
White people	54.20	129
Black people	42.86	102
Others	2.94	7
**Gender**		
Male	39.92	95
Female	60.08	143
**Education**		
None	3.36	8
Primary	24.79	59
Matric/beyond	71.85	171
**Marital status**		
Single	15.97	380
Married	56.30	134
Divorced	5.46	13
Widowed	21.85	52

Only 43.28% (*n* = 103) of participants had a comorbidity with hypertension and of these 63.64% did not consider their hypertension more burdensome than the other comorbidity (Table 2).

**TABLE 2:** Hypertension and comorbidities.

Variable	Frequency	%	CI
**Comorbidity**			
No	135	56.72	50.17–63.11
Yes	103	43.28	36.89–49.83
Total	238	100.00	-
**Report of hypertension problem**			
No	63	63.64	53.36–73.07
Yes	35	35.35	26.01–45.60
Total	98	100.00	-

CI, confidence interval.

The proportions of participants with controlled and uncontrolled BPs are shown in Table 3 and indicate that only 40.34% and 39.92% were controlled to targets at current visit and in the last 3 months, respectively. Participants who were black people and divorced were significantly more likely to have a higher SBP. However, participants who were single and male were significantly more likely to have a higher DBP.

**TABLE 3:** Blood pressure control (*n* = 239).

Variable	%	*n*	CI
**BP control (all visits)**			
Uncontrolled	60.08	143	53.56–66.36
Controlled	39.92	95	33.64–46.44
Total	100.00	-	-
**BP control_(current visit)**			
Uncontrolled	59.66	142	53.13–65.95
Controlled	40.34	96	34.05–46.87
Total	100.00	-	-

BP, blood pressure; CI, confidence interval.

The mean treatment duration was 113.8 months (Table 4). Participants who were widowed (*p* < 0.01), white (*p* = 0.03), aged 45–64 years (*p* = 0.04) and female (*p* = 0.04) were significantly more likely to have longer mean durations of treatment. There was no significant difference in the duration of treatment between participants with controlled BP and those uncontrolled. There was no significant association between sociodemographic characteristics and BP control.

**TABLE 4:** Blood pressure control, sociodemographics and mean treatment duration.

Variables	*n*	Mean (months)	s.d.	*p*
**Total participants**	239	113.80	106.60	
Uncontrolled BP	143	112.66	101.14	0.08
Controlled BP	95	115.42	114.87	-
**Marital status**				
Single	38	91.58	93.91	0.00*
Married	134	112.31	108.53	-
Divorced	13	127.00	130.00	-
Widowed	52	132.12	103.93	-
**Race**				
White people	129	130.61	108.66	0.03*
Black people	102	93.62	101.53	-
Others	7	96.86	100.66	-
**Age (years)**				
25–34	12	50.75	67.66	0.01*
35–44	28	85.07	68.86	-
45–54	70	95.39	107.59	-
55–64	60	132.13	105.65	-
65–74	53	137.70	109.19	-
75–84	15	145.40	140.89	-
**Sex**				
Male	95	96.32	82.46	0.04*
Female	143	125.35	118.86	-

BP, blood pressure; s.d., standard deviation.

* *p* < 0.05 statistically significant.

The BOT scores are shown in Table 5. Most of the participants (75%) reported a low TBOT. The mean TBOT was 19.7 (out of a possible score of 140). Regarding the relationship between TBOT/BOT and BP control, TBOT was not significantly different amongst sociodemographic groups (Table 6). However, participants with uncontrolled BP were significantly more likely to have a higher score in BOT1 component (*p* = 0.04). Except for single participants who were significantly more likely to score higher than others (*p* < 0.01), there were no significant differences in TBOT scores across other sociodemographic groups. On further analyses, race was found to be significantly associated with item 3 of the TBQ that enquired how problematic was ‘the efforts you make not to forget to take your medications’ (*p* = 0.006). Similarly, age and marital status were significantly associated with item 11 (The financial burden associated with your healthcare [e.g. out-of-pocket expenses not covered by insurance] of the TBQ that enquired how problematic was ‘the financial burden associated with your healthcare’) (*p* = 0.007 and 0.034, respectively).

**TABLE 5:** Burden of treatment components.

Overall	Mean	
	%	*n*
Mean BOT score	19.7 (of a possible score of 140)	
**BOT related to medication regimen**		
None	15.25	36
Low	75.00	177
Moderate	9.75	23
High	0	-
Total	100.00	-
**BOT related to health system navigation**		
None	43.22	102
Low	41.10	97
Moderate	14.83	35
High	0.85	2
Total	100.00	-
**BOT related to lifestyle changes and support**		
None	22.55	53
Low	48.94	115
Moderate	22.98	54
High	5.53	13
Total	100.00	-

BOT, burden of treatment.

**TABLE 6:** Association between burden of treatment and blood pressure control.

BOT*Control	Mean BOT	s.d.	*p*
TBOT*Control			
Uncontrolled BP	20.3357	20.0955	0.53
Controlled BP	18.7053	19.3065	-
BOT1*Control Issues around medication regimen			
Uncontrolled BP	2.9441	5.7712	0.04
Controlled BP	1.6316	3.0459	-
BOT2*Control Navigating healthcare system			
Uncontrolled BP	6.0699	8.4016	0.43
Controlled BP	6.9579	8.7238	-
BOT3*Control Life-style modification and support			
Uncontrolled BP	11.3636	10.3920	0.46
Controlled BP	10.2842	11.8534	-

BP, blood pressure; BOT, burden of treatment; s.d., standard deviation; TBOT, total BOT.

## Discussion

As far as the authors are aware, this is the first study to assess BOT and determine its relationship with hypertension control in South Africa. We found that most participants had suboptimal hypertension control (60%) but reported none or low TBOT (90%). Comorbidity was common amongst participants (43.3%) and amongst this group most (63.6%) did not consider hypertension to be more burdensome than the other health problems. Although there was no significant association between TBOT and hypertension control, participants with uncontrolled BP were significantly more likely to report higher scores in BOT associated with issues around medication regimen than those with controlled BP (*p* = 0.04).

The finding of prevalent suboptimal hypertension control in this study reiterates those of previous studies in South Africa and elsewhere,^4,5^ and highlights the need to develop a scalable and sustainable intervention plan that targets factors that have been associated with poor BP control, including poor lifestyle behaviours (smoking, diet, alcohol, obesity, etc.), weak social support network and high BOT as responsible factors.^4,5,6^ However, the current study finds contrary on BOT and suggests that high BOT may not play a significant role in hypertension control in the South African PHC setting. Rather, addressing problems related to medication regimen such as poor access to anti-hypertensives, irrational prescriptions secondary to poor healthcare providers’ adherence to evidence-based guidelines and poor patient adherence to medications and lifestyle changes may be key to improving hypertension control in this setting.^27^ Medication-related problems often arise from fragmentations within the health system, complex drug regimen, poor communication between the clinician and the patient, patient’s physical or cognitive limitations, socioeconomic status and behaviours that make patients forget, run out or take their medications wrongly.^27^ Efforts to address poor patient adherence must build on a robust doctor–patient relationship and respond to individual patient’s reason(s) for non-adherence, be sensitive to non-verbal cues, motivate the patient towards desired behaviours, take cognisance of the cognitive capacity of the individual patient, confirm patient’s understanding of the issues and have a reminder system.^27^ Total BOT is a composite measure, and the finding of a low BOT score and lack of association with BP control are counter-intuitive in that these contradict reports of financial, health system and social barriers to accessing PHC services in South Africa. These barriers include long queues and transit times, high patient load, unavailability of medications, poor staff attitudes and poor physical access.^28,29^ However, there are several possible explanations for this: firstly, medical services at the PHC level are free in South Africa^30^ and may remove significant barriers to accessing care, including for hypertension. Secondly, significant strides have been made by the South African government to ensure that PHC clinics are located within 5 km of most residential areas and this is the case in the research setting. Thirdly, the research setting was peri-urban, and most participants were white and had high school certificate or higher – characteristics that are known to increase patient assertiveness and engagement with healthcare providers,^31,32^ and may account for the reported low burden scores with navigating the health system.

Fourthly, hypertension is symptomless (even when poorly controlled)^2^ and patients may not perceive a need for social support until complications that are physically debilitating ensue. In addition, most of the participants (56.30%) in this study were married or cohabiting and were likely to enjoy the benefits of social companionship. These may make participants report low BOT scores because social companionship ameliorates stress inherent in living alone, mitigates high BOT and improves treatment adherence and outcomes.^5,6,17^ Not surprisingly, in this study, divorced and single participants were significantly more likely to have a higher mean SBP and DBP, respectively. However, this study did not find any association between BP control and marital status, which may imply that there may be factors within the marriage institution such as social support whose absence or presence drives this association rather than the marriage itself. Nonetheless, where social companionship is lacking, such as in the elderly with hypertension who live alone, community-based health peer groups and home visits by community health workers could be used to promote social and treatment support, ameliorate BOT and consequently improve BP control.

Comorbidity was common in this study; previous multi-morbidity studies and BOT outside our context reported it to impose a high BOT, worsen treatment adherence and result in poor disease control.^1,2,3,4,5^ There is a need for BOT studies and multi-morbidity to be carried out in our context. However, this highlights the need for healthcare professionals to screen for co-existing illnesses and BOT, not only for clinical risk stratification and treatment.^16^ If a high BOT is found, resources for social and treatment support need to be recruited. However, healthcare professionals do not often respond to poor disease by assessing BOT.^16,19^ Rather, they escalate and make treatment regimens more complex.^16,19^ Clinicians should aim to understand the impact of the BOT imposed on their patients by the physical demands of the disease and comorbid condition(s), the complexity of drug prescriptions and the need for self-care. Reflecting on these may result in healthcare professionals being more patient-centred and rational in their prescribing behaviour.

### Limitations of the study

This study has potential limitations: firstly, although the BOT questionnaire has a high internal validity, it was developed in France and may not adequately address issues found in the African psychosocial contexts. Disruptive effects of treatment on working and social life, economic and time losses of clinic visits and the burden associated with the learning of new skills for coping and self-care are not currently measured by the TBQ. Secondly, the location of the study facility in a predominantly white area and the decanting programme that back-referred patients to their areas of residence during the study led to under-representation of black people, Asians and mixed race. The generalisation of study findings nationally or elsewhere therefore needs to be done with caution. Thirdly, the exclusion of the patients who presented after-hours or on weekends because they work and may therefore have higher BOT relating to navigating the health system, those who were in the home drug delivery programme (Kgatelopele Programme) and needed to have controlled BP to qualify, and those eligible patients who were managed in other clinics (such as the HIV clinic) could have introduced selection bias, which would have resulted in errors in BP control and BOT estimates. Lastly, BOT is dynamic and the findings of a cross-sectional study such as this may not apply to other times, even in the same patient. Notwithstanding these limitations, this study adds to the body of knowledge on BOT by providing the first insight ever into the magnitude of BOT amongst patients with hypertension in South African PHC and its influence on BP control. This study therefore lays the foundation for further research on this concept in South Africa.

## Conclusion

The TBOT is low amongst patients with hypertension and does not have significant association with BP control. However, the association of poor BP control with higher BOT1 scores highlights the need for strengthening healthcare providers’ adherence to evidence-based guidelines, simplifying drug regimen and promoting patient adherence to treatment in South African PHC. The limitations of this study necessitate that longitudinal and nationally representative quantitative studies on BOT and BP control are required. Qualitative studies are also needed to gain an in-depth understanding of BOT, especially in the African context. The escalation of TBOT and adverse outcomes with comorbidity requires the inclusion of multi-morbid patients in these studies. Because the TBQ was adopted from a developed country, there is a need for tests of validation and appropriateness in African context.

## References

[1] Cappuccio FP, Miller MA Cardiovascular disease and hypertension in sub-Saharan Africa: Burden, risk and interventions. Intern Emerg Med. 2016;11:299–305. 10.1007/s11739-016-1423-9 27001886 PMC4820479

[2] World Health Organization A global brief on hypertension. Silent killer, public, public health crises. WHO/DCO/WHD/2013.2. Geneva: WHO press; 2013.

[3] Statistics South Africa South African demographic and health survey, key indicators report Blood pressure status page 48. Report 03-00-092016(1), Pretoria: National Department of Health, Civitas Building; 2016.

[4] Batubenga MM, Omole OB, Bondo MC Factors associated with blood pressure control among patients attending the outpatient clinic of a South African district hospital. Trop Doct. 2015;45(4):225–230. 10.1177/0049475515587160 26002722

[5] Onwukwe SC, Omole OB Drug thrapy, lifestyle modification and blood pressure control in a primary care facility, south of Johannesburg, South Africa: An audit of hypertension management. S Afr Fam Pract. 2012;54(2):156–161. 10.1080/20786204.2012.10874196

[6] Cornwell EY, Waite LJ Social network resources and management of hypertension. J Health Soc Behav. 2012;53(2):215–231. 10.1177/0022146512446832 22660826 PMC3727627

[7] Menanga A, Edie S, Kingue S Factors associated with blood pressure control amongst adults with hypertension in Yaounde, Camerron: A cross sectional study. Cardiovasc Diagn Ther. 2016;6(5):439–445. 10.21037/cdt.2016.04.03 27747167 PMC5059397

[8] Paulsen MS, Andersen M, Thomsen JL, Schroll H, Larsen PV, Lykkegard J Multimorbidity and blood pressure control in 37651 hypertensive patients from Danish general practice. J AM Heart Assoc. 2012;2:e004531 10.1161/JAHA.112.004531 23525411 PMC3603256

[9] Asgary R, Sckell B, Alcabes A, Naderi R, Schoenthaler A, Ogedegbe G Rates and predictors of uncontrolled hypertension among hypertensive homeless adults using new york city shelter-based clinic. Ann Fam Med. 2016;14(1):41–46. 10.13370/afm.1882 26755782 PMC4709154

[10] Karmacharya BM, Koju RP, Logerfo JP, Fitzpatrick AL Awareness treatment and control of hypertension in Nepal:findings from the Dhulikhal heart study. BMJ. 2016;9(1):1–8. 10.1136/hearttasia-2016-010766 PMC5237714 28123454

[11] Cheong AT, Sazlina SG, Tong SF, Azah AS, Salmiah S Poor blood pressure control and its associated factors among older people with hypertension: A cross-sectional study in six public primare care clinics in Malaysia. Malays Fam Physician. 2015;10(1):19–25. PMC4567889 26425291

[12] Adeniyi OA, Yogesweran P, Longo-Mbenza B, Goon TG Uncontrolled hypertension and its determinants in patients with concomittant type 2 diabetes mellitus(T2DM). PLoS One. 2016;11(3):e0150033 10.1371/Journal.pone.0150033 26930050 PMC4773066

[13] May CR, Eton DT, Boehmer K, et al Rethinking the patient: Using burden of treatment theory to understand the changing dynamics of illness. BMC Health Serv Res. 2014;14:281 10.1186/1472-6963-14 24969758 PMC4080515

[14] Demain S, Gonçalves A, Areia C, et al Living with, managing and minimising treatment burden in long term conditions: A systematic review of qualitative research. PLoS One. 2015;10(5):e0125457 10.1371/journal.pone.01 26024379 PMC4449201

[15] Eton DT, Ridgeway JL, Egginton JS, et al Finalizing a measurement framework for the burden of treatment in complex patients with chronic conditions. Patient Relat Outcome Meas. 2015; 6:117–126. 10.2147/PROM.S78955 25848328 PMC4383147

[16] Eton DT, Ramalho de Oliveira D, Egginton JS, et al Building a measurement framework of burden of treatment in complex patients with chronic conditions: A qualitative study. Patient Relat Outcome Meas. 2012;3:39–49. 10.2147/PROM.S34681 23185121 PMC3506008

[17] Gallacher K, May CR, Montori VM, Mair FS Understanding patients’ experiences of treatment burden in chronic heart failure using normalization process theory. Ann Fam Med. 2016;9(3):235–243. 10.1370/afm.1249 PMC3090432 21555751

[18] Gallacher K, Jani B, Morrison D, et al Qualitative systematic reviews of treatment burden in stroke, heart failure and diabetes – Methodological challenges and solutions. BMC Med Res Methodol. 2013;13:10 10.1186/1471-2288-13 23356353 PMC3568050

[19] Ridgeway JL, Egginton JS, Tiedje K, et al Factors that lessen the burden of treatment in complex patients with chronic conditions: A qualitative study. Patient Prefer Adherence. 2014;8:339–351. 10.2147/PPA.S58014 24672228 PMC3964167

[20] Gallacher K, Morrison D, Jani B, et al Uncovering treatment burden as a key concept for stroke care: A systematic review of qualitative research. PLoS Med. 2013;10(6):e1001473 10.1371/journal.pmed.1 23824703 PMC3692487

[21] Tran VT, Barnes C, Montori VM, et al Taxonomy of the burden of treatment: A multi-country web-based qualitative study of patients with chronic conditions. BMC Med. 2015;13:115 10.1186/s12916-015-03 25971838 PMC4446135

[22] Mohammed MA, Moles RJ, Chen TF Medication-related burden and patients’ lived experience with medicine: A systematic review and metasynthesis of qualitative studies. BMJ Open. 2016;6(2):e010035 10.1136/bmjopen-2015 PMC4746464 26839015

[23] Tran VT, Montori VM, Eton DT, et al Development and description of measurement properties of an instrument to assess treatment burden among patients with multiple chronic conditions. BMC Med. 2012;10:68 10.1186/1741-7015-10 22762722 PMC3402984

[24] Tran V, Harrington M, Montori V, Wicks P, Revaud P Adaption and validation of the treatment burden questionnaire(TBQ) in English using an internet paltform. BMC Med. 2014;12:109 10.1186/1741-7015-12-109 24989988 PMC4098922

[25] Gupta AK Rascial differences in response to anti hypertensive therapy: Does one size fit all? Int J Prev Med. 2010;1(4):217–219. 21566775 PMC3075515

[26] Seedat YK, Rayner BL, Veriava Y South African hypertension guideline 2014. Cardiovasc J Afr. 2014;25(6):288–294. 10.5830/CVJA-2014-062 25629715 PMC4327181

[27] Tsiantou V, Pantzou P, Kryrioplus J Factors affecting adherence to antihypertensive medication in Greece: Results from a qualitative study. Patient Prefer Adherence. 2010;4:335–343. 20859460 10.2147/ppa.s12326 PMC2943225

[28] Daniels S, Zweigenthali V, Reagan G Assessing the impact of a waiting times on survey on reducing waiting times in urban primary care clinics in Cape Town, South Africa. J Public Health Afr. 2017;8(1):639 10.4081/Jphia.2017.639 29218136 PMC5708303

[29] National Department of Health Towards qualitycare for patients. National core standards for health establishments in South Africa [homepage on the Internet]. Tshwane: Department of Health; 2011 [cited 2019 Mar 24]. Available from: www.health.gov.za.

[30] National Department of Health Uniform patient fee schedule – Classification of patients [homepage on the Internet]. 2018 [cited 2019 Mar 24]. Available from: www.health.gov.za>index.php>catetegory.

[31] Bell CN, Thorpe Jnr RJ, Laveij TA Race/ethnicity and hypertension: The role of social support. AM J Hypertens. 2010;23(5):534–540. 10.1038/ajh.2018.28 20186126 PMC3102008

[32] Cois A, Ehrlich R Analysing the socioeconomic determinants of hypertension in South africa. BMC Public Health. 2014;14:414 https://doi.org/10-1186/1471-2458-14-414 24885860 10.1186/1471-2458-14-414 PMC4021547

